# *RDWN6*^*XB*^, a major quantitative trait locus positively enhances root system architecture under nitrogen deficiency in rice

**DOI:** 10.1186/s12870-018-1620-y

**Published:** 2019-01-08

**Authors:** Galal Bakr Anis, Yingxin Zhang, Anowerul Islam, Yue Zhang, Yongrun Cao, Weixun Wu, Liyong Cao, Shihua Cheng

**Affiliations:** 1 Key Laboratory for Zhejiang Super Rice Research and State Key Laboratory of Rice Biology, China National Rice Research Institute, Hangzhou 310006, China; 2 Rice Research and Training Center, Field Crops Research Institute, Agriculture Research Center, Kafrelsheikh, 33717 Egypt

**Keywords:** Rice, *qRDWN6*^*XB*^, Quantitative trait locus, Nitrogen deficiency tolerance

## Abstract

**Background:**

Nitrogen (N) is a major input cost in rice production, in addition to causing severe pollution to agricultural and ecological environments. Root dry weight has been considered the most important component related to crop yields than the other root traits. Therefore, development of rice varieties/lines with low input of N fertilizer and higher root traits are essential for sustainable rice production.

**Results:**

In this context, a main effect quantitative trait locus *qRDWN6*^*XB*^ on the long arm of chromosome 6 which positively confers tolerance to N deficiency in the *Indica* rice variety XieqingzaoB, was identified using a chromosomal segment substitution line (CSSL) population. *qRDWN6*^*XB*^ was determined to be located near marker InD90 on chromosome 6 based on association analysis of phenotype data from three N levels and 120 polymorphic molecular markers. The target chromosomal segment substitution line CSSL45, which has the higher root dry weight (RDW) than *indica* cultivar Zhonghui9308 and carry *qRDWN6*^*XB*^*,* was selected for further study. A BC_5_F_2:3_ population derived from a cross between CSSL45 and Zhonghui9308 was constructed. To fine-map *qRDWN6*^*XB*^, we used the homozygous recombinant plants and ultimately this locus was narrowed to a 52.3-kb between markers ND-4 and RM19771, which contains nine candidate genes in this region. One of these genes, *LOC_Os06g15910* as a potassium transporter was considered a strong candidate gene for the *RDWN6*^*XB*^ locus.

**Conclusions:**

The identification of *qRDWN6*^*XB*^ provides a new genetic resource for breeding rice varieties and a starting point to improve grain yield despite the decreased input of N fertilizers. The newly developed and tightly linked InDel marker ND-4 will be useful to improve the root system architecture under low N by marker-assisted selection (MAS) in rice breeding programs.

## Background

Rice (*Oryza sativa* L.) is one of the most important three major cereal crops for billions of people, most of who live in rural and urban areas of tropical and subtropical Asian countries [1, 2]. Therefore, rice production is playing an important role in ensuring food security and poverty alleviation in rice-eating countries in addition farmers’ incomes for the majority of the world population [3]. According to previous estimates, the world’s population will reach 8 billion by 2030 [4], and 9 billion by 2050 [5]. The dramatically increasing population will be exhausted the sustainable agricultural production system, therefore rice production must be increased by 50% in order to meet the growing demand and food needs in the future [4].

Nitrogen (N) is the most essential nutrient plant need in crop developments and high quantity. It is often the most yield-limiting nutrient in rice crop production in many countries [6, 7]. Nitrogen improves rice grain yield and grain quality by improving; root system, panicle numbers, leaf area, filled grains numbers, thousand grain weight, grain formation, and protein synthesis [8]. Of the total rice production costs, nitrogen fertilizers are major input cost and cause environmental pollution in case of excessive quantities used [9]. The problems and negative environmental effects of excessive nitrogen (N) quantities are well documented [10–12]. Nitrogen (N) pollution from fertilizers is the biggest environmental disaster affecting human health in multiple ways. Therefore, it is necessary to develop new rice lines/varieties with improved N use efficiency and high responses to N deficiency stress for rice breeding programs and sustainable rice production [13, 14]. Nitrogen deficiency conditions lead to a reduction in many agronomic traits and, as a result, growth rate, grain and biomass yield decrease [15]. Additionally, most of the physiological processes, nitrogen metabolism, carbon metabolism, hormone metabolism and photosynthetic, are negatively affected [16]. Thus, developing rice genotypes with high tolerance to N deficiency is a highly desired characteristic for sustainable rice production [17].

Root traits are the important nitrogen-deficiency tolerance measurements, water uptake and as the major interface between rice plant and various biotic and abiotic stresses in natural soil [18]. Numerous studies have measured root traits as the ratio of the trait values under low nitrogen to those under normal, with a criterion for selecting genotypes for the tolerance of low nitrogen. Root number, root length, root volume, root thickness, dry weight of roots, dry weight of the shoot and the total dry weight, are important traits for studying of nitrogen-deficiency tolerance in rice breeding programs. Studying like these traits is difficult to handle in soil environment. To meet these limitations, many of studies grow rice seedlings in hydroponic system using diverse mapping populations to identify the QTLs responses to nitrogen deficiency tolerance in rice [19–21]. Examination of N deficiency tolerance in rice using hydroponic systems as controlled conditions is an alternative approach.

Several quantitative trait loci (QTLs) associated with nitrogen-deficiency tolerance in rice were detected in the field and greenhouse experiments using diverse mapped populations, e.g. in Chromosomal Segment Substitution Lines [22–24], Recombinant Inbred Lines [19, 25], Introgression Lines [26] and Backcross Recombinant Lines [27]. Feng et al. [28] detected seven QTLs associated with N-deficiency tolerance on chromosomes 1, 2, 3 and 8 at seedling stage. Zhao et al. [29] mapped 28 QTLs under two N levels and 16 QTLs of their relative traits for seedling traits related to low nitrogen tolerance in rice. Main effect QTLs on chromosome 3 were mapped using a DH population under three nitrogen conditions [30]. Obara et al. [20] mapped *qRL6.1,* a major QTL on chromosome 6 for root elongation of rice seedling under different N levels. Many genes associated with utilization of nitrogen (N) for root seedling traits have been identified in rice [31–33]. Studying and understanding the mechanisms of rice N utilization at a molecular level may help to improve rice varieties for N deficiency tolerance.

The super hybrid rice ‘Xieyou9308’ is one of the earliest hybrids occupied greater areas in China [34]. Building on our previous study, some QTLs were identified under N deficiency using a set of chromosomal segment substitution lines (CSSLs) derived from XieqingzaoB/Zhonghui9308 as two parents of the hybrid ‘Xieyou9308’. Here in this study, the objectives were to validate and fine map a major QTL, *qRDWN6*^*XB*^, for root dry weight under N deficiency using F_2:3_ populations derived from a cross between the target CSSL45 and genetic background parent Zhonghui9308.

## Methods

### Rice materials and fine mapping population

**Fig. 1:**
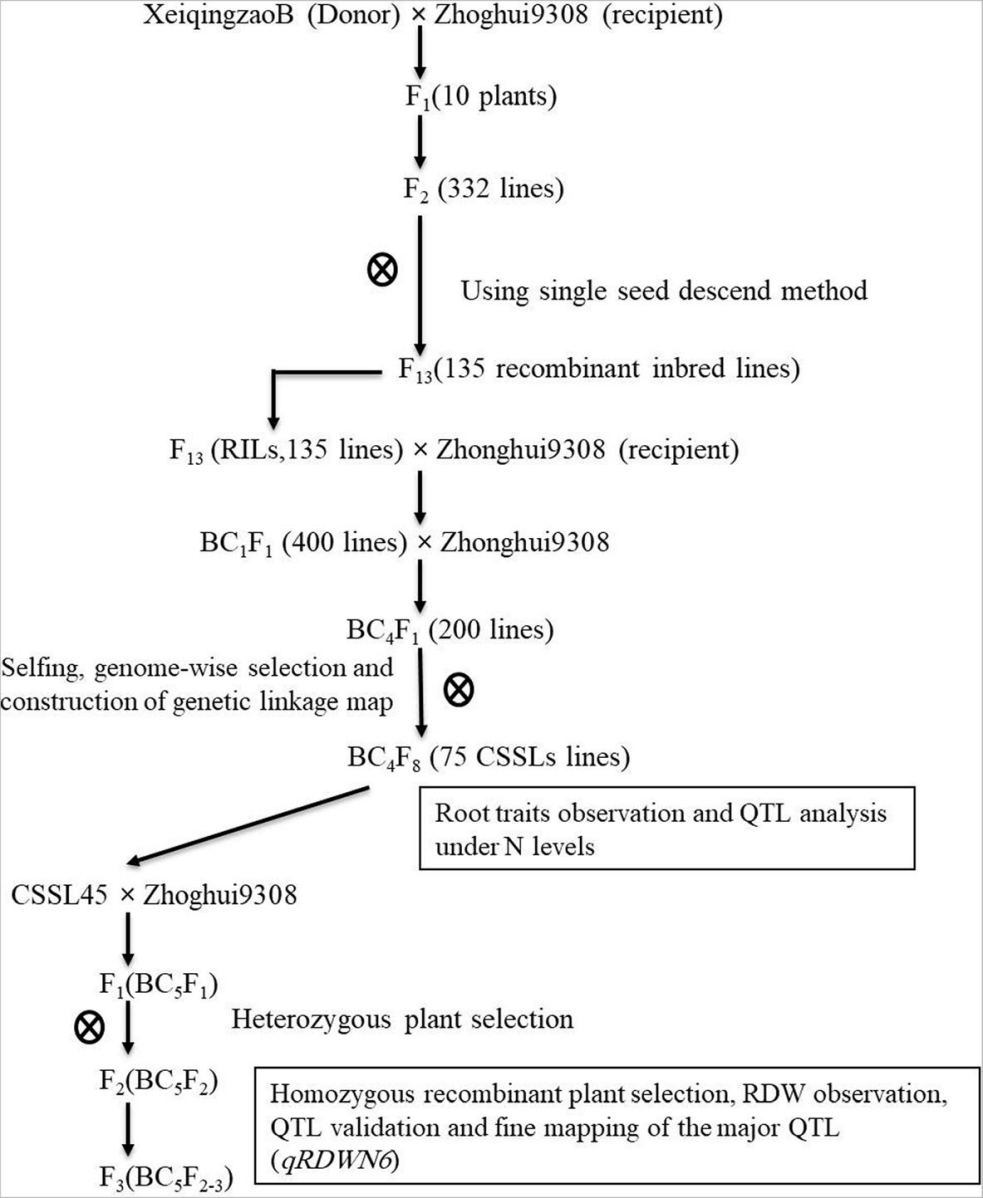
The breeding strategy applied for QTL and fine mapping in this study

In our previous study, a set of 75 Chromosome Segment Substitution Lines (CSSLs) were evaluated for root dry weight and other related root traits under N deficiency at the seedling. The donor parent XieqingzaoB (XQZB) and recipient parent Zhonghui9308 (ZH9308) as parental lines of this population exhibited high and low of RDW and other related root traits, respectively, under N deficiency [24]. For this study, one line of CSSLs population, namely CSSL45, which showed high root dry weight (RDW) values compared with Zhonghui9308 under N^−^ deficiency and harboring the *qRDWN6*^*XB*^ a root dry weight conditioning locus, was crossed with the genetic background parent Zhonghui9308, and a total of 2200 F_2:3_(BC_5_F_2:3_) seedlings obtained from the cross were evaluated to perform fine mapping of the *qRDWN6*^*XB*^ locus under low nitrogen on chromosome 6 as reported by our previous study [24]. Therefore, XieqingzaoB, Zhonghui9308, CSSL45 and F_2:3_(BC_5_F_2:3_) population derived from a cross between CSSL45/Zhonghui9308 were used in this study. The development scheme of rice populations used here is shown in Fig. 1.

### Growth conditions in hydroponic culture

**Fig. 2:**
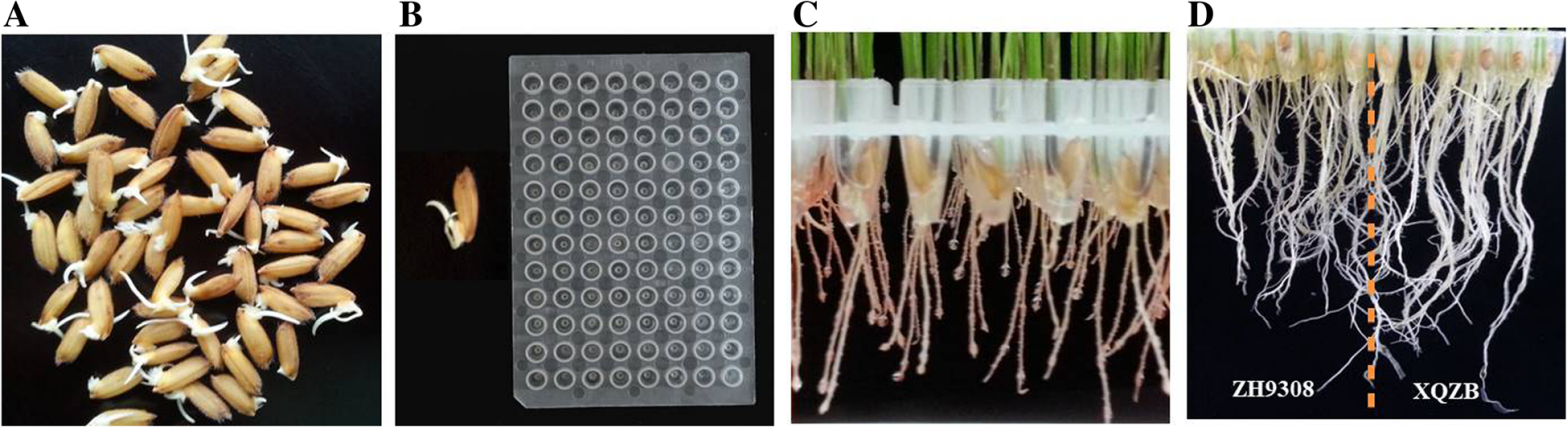
Seed germination, hydroponic method and root phenotypes. (**a**) Rice seeds were germinated in incubator for 3 days. (**b**) After 72 h, healthy germinated seeds were transplanting into eppendorf tubes. (**c**) Planting for one week before starting exposure to the solution. (**d**) Image for root phenotypes taken from the seedling stage for ZH9308 and XQZB under N deficiency

Seeds of XieqingzaoB, Zhonghui9308, CSSL45 and F_2:3_(BC_5_F_2:3_) populations were soaked in sterilized distilled water for 2 days at 30 °C and then incubated at 29 °C for 24 h (Fig. 2a). The healthy germinated seeds were transplanting into Eppendorf tubes for 1 week before starting exposure to the solution (Fig. 2b, c). The experiment was conducted at China National Rice Research Institute (CNRRI) at Hangzhou, China, during 2017 and 2018. For confirming and comparison of root dry weight and other related root traits of XieqingzaoB, Zhonghui9308 and CSSL45, fifteen seedlings of these parental lines were evaluated under both (N^+^) normal and (N^−^) deficiency conditions. Yoshida rice nutrient solution was used with some minor modifications [35]. For nitrogen (N^−^) deficiency condition, the concentration of (NH_4_)_2_SO_4_ decreased to 0.42 (mg/l). The nutrient solution was prepared in distilled water and renewed every 5 days. The plants were grown and screened in the chamber with a 30 ± 3/21 ± 2 °C day/night temperature and 70% relative humidity. The F_2:3_(BC_5_F_2:3_) plants were evaluated only under nitrogen deficiency (N^−^) treatment, and root dry weight under nitrogen deficiency (N^−^) was measured as RDWN. The root dry weight and other related traits were recorded after 5 weeks in a chamber (Fig. 2d).

### Sampling and measurements

At seedling stage and after 5 weeks from growing, plants were sampled for trait measurements and genotypic analysis. Multiple root traits including root number, root length, shoot length, shoot dry weight, root dry weight and total dry weight of parental lines (XieqingzaoB, Zhonghui9308 and CSSL45) were measured under both (N^+^) normal and (N^−^) deficiency conditions. Root dry weight of F_2:3_(BC_5_F_2:3_) plants was measured as the weight of root mass after 2 days of drying at 70 °C.

### DNA extraction and development of molecular markers

DNA was extracted from the fresh leaves of each plants of F_2:3_ (BC_5_F_2:3_) population and their parents using the CTAB method described by Luo et al. [36]. PCR protocol was performed in a 10-μL reaction volume containing 1.5 μL of 20.0 ng/μL template genomic DNA, 1.0 μL of 10× PCR buffer, 0.25 μL of 1.0 pmol/μL dNTPs, 1.5 μL of 2.0 pmol/μL primer pairs, 0.06 μL of 5.0 U/μL Taq DNA polymerase and 5.69 μL of ddH_2_O. The amplification protocol consisted of an initial denaturation step (94 °C for 5 min), followed by 32 cycles of 94 °C for 30 s, 55 °C for 30 s, and 72 °C for 1 min. The reactions were completed with a final extension step of 72 °C for 7 min. The PCR products were separated by electrophoresis on 8% non-denaturing polyacrylamide gels and then visualized by silver staining [37]. New InDel markers in the specific genomic region were designed according to genome sequence differences between the *indica* cv. XieqingzaoB and the *indica* cv. Zhonghui9308. The sequences of these new markers used in this investigation are listed in Table 1.

**Table 1:** DNA primers sequences for polymorphic markers designed for fine mapping of *qRDWN6*^*XB*^ on chromosome 6

Marker	Marker type	Forward primer sequence	Reverse primer sequence	Purpose	Size
RM5963	SSR	TCAAGTTACGGGAAATGTGTGG	CTGCCTAGCTTCCGTTTCTCC	Primary mapping	139
InD90	InDel	CCTCATCCAGGGGTCATGTA	CGGTCAAGTGTCATCCAGGT	Primary mapping	19
RM20069	SSR	GCGAGCGAGAGGAGAGATAGACG	CGAATTCGGCACGAGTAATAGGG	Primary mapping	157
ND-3	InDel	AGACGGTGATATCGGTGAGT	GGAGTTTAGTGGCTGCATCA	Fine mapping	258
ND-4	InDel	AAAACACCAAAGAATCCGGC	AGGATAGGAAAACCGTGCAA	Fine mapping	282
ND-5	InDel	GCTTTAGCTACGGTTTCCGA	TTTGACTCGTCCCAATAGGC	Fine mapping	225
ND-11	InDel	CCGAGTAGCGAAGCTCAAAT	CTAGCATGGACGAACGGATG	Fine mapping	103
ND-13	InDel	ATTCAGCGTTCCTTAACCCG	GACAGAGTCGAGAAACCGTG	Fine mapping	116
ND-15	InDel	AGATCTCACATGATTATATTCCGA	TCTGCAACAAAGTGAAATCCT	Fine mapping	117

*SSR* Simple Sequence Repeat, *InDel* Insertion/deletion

### Statistical analysis of data

Mean phenotypic values for seedling root traits were compared using the Student’s *t*-test. Previously, a total of 120 SSR and Insertion/Deletion markers were distributed along the rice genome and used for identifying the IciMapping QTLs in 75 CSSLs [24]. Phenotypic correlations were calculated using a generalized linear model implemented within the SAS statistical software package.

## Results

### Characterization of XQZB and ZH9308 under different N levels

**Fig. 3:**
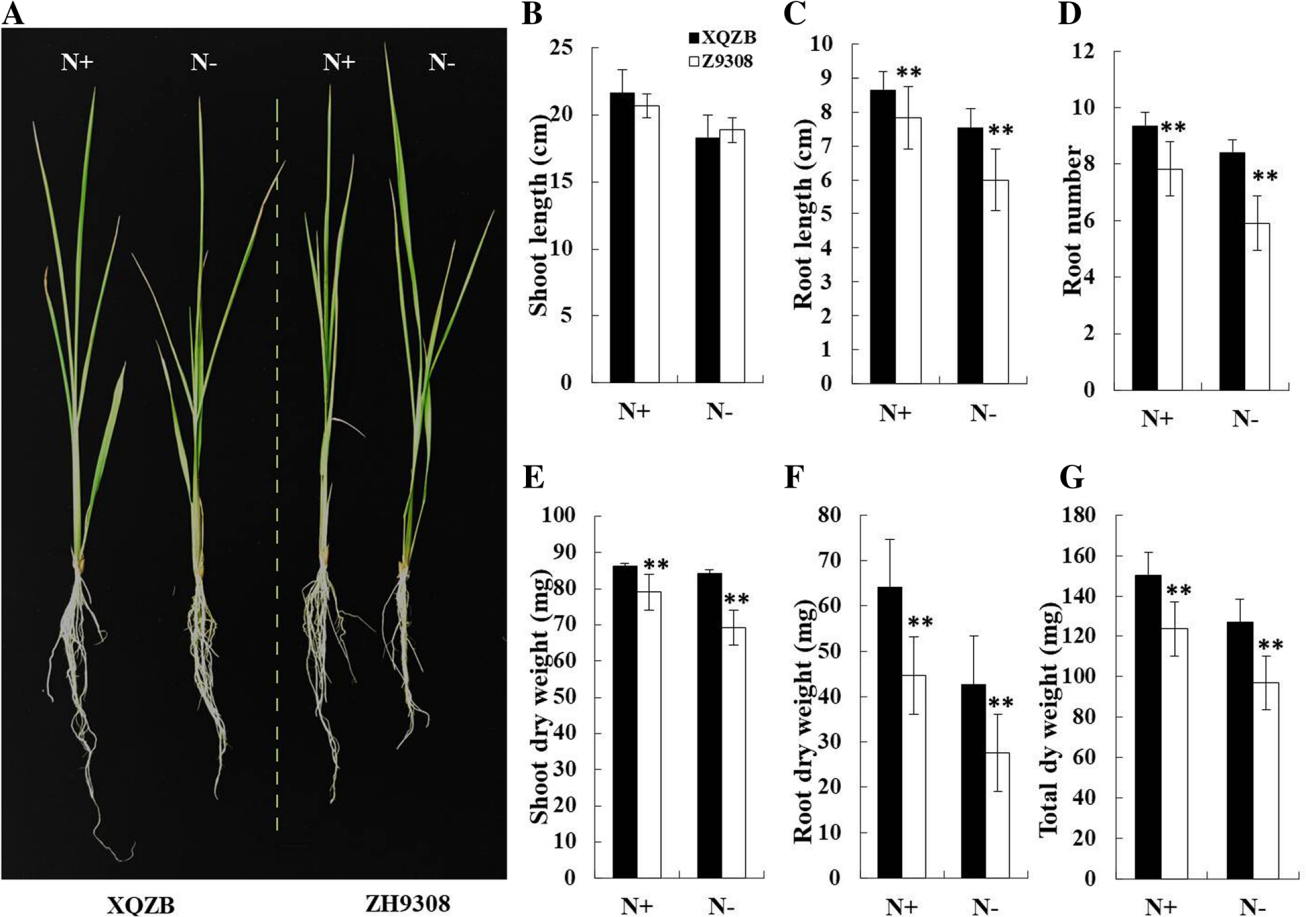
Phenotypic characterization of XQZB and ZH9308. (**a**) Comparison of XQZB and ZH9308 at seedling stage under both N^+^ and N^−^ levels. (**b**, **c**, **d**, **e**, **f** and **g)** Comparison of shoot length, root length, root number, shoot dry weight, root dry weight and total dry weight between XQZB and ZH9308 under both N^+^ and N^−^ levels. Five plants (*n* = 5) were used to measure the growth parameters. The asterisks represent statistical significance between XQZB and ZH9308, determined by a student’s *t*-test (**P < 0.01)

To identify the major QTLs responsible for nitrogen deficiency tolerance in rice, a set of 75 CSSLs population derived from an *Indica* c.v XieqingzaoB as the donor parent and *Indica* c.v Zhonghui9308 as the recurrent parent were used [24]. The phenotypic values for six seedling root traits of XieqingzaoB and Zhonghui9308 under both low and normal N are shown in Fig. 3. There were significant differences between XieqingzaoB and Zhonghui9308 for all studied root traits (root number, root length, root dry weight, shoot dry weight and total dry weight). The two parents, XieqingzaoB and Zhonghui9308 exhibited no differences in their shoot length under both N levels (Fig. 3b). Zhonghui9308 was significantly inhibited reductions in most of the root traits compared with XieqingzaoB under N^−^ deficiency level. The donor parent XieqingzaoB showing consistently higher values than Zhonghui9308 in two N conditions, thereby suggesting that Zhonghui9308 showed a smaller response to N deficiency than XieqingzaoB (Fig. 3).

### Correlation analysis among root traits

**Fig. 4:**
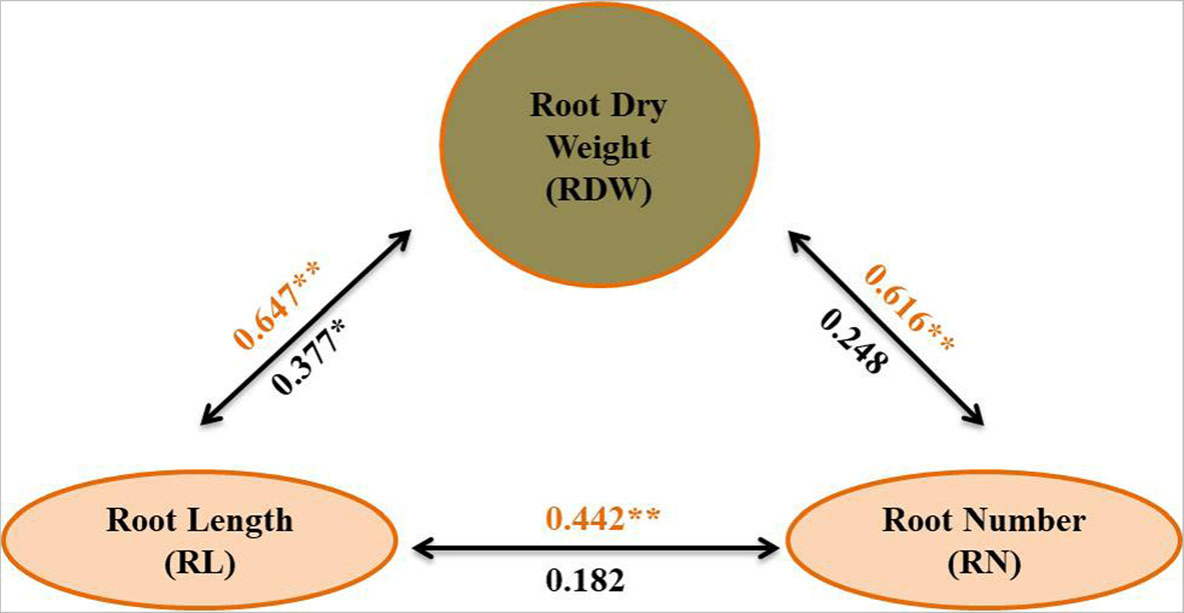
Phenotypic correlations among root dry weight and other related traits. The *red* and *black* numbers represent Person correlation coefficients between the traits under N^−^ and N^+^, respectively. Single and double asterisk represent statistical significant at *P* < 0.05 and 0.01, respectively

Phenotypic correlations coefficient analysis between root dry weight and other related root traits like root length and root number under both N conditions were performed (Fig. 4). Data by red color correspond to correlations under low N^−^ level and data by black color correspond to correlations under normal N^+^ level. As shown in Fig. 4, three phenotypic seedling root traits were significant and highly significant correlation coefficients across two N levels except for correlations between root dry weight and root number, and between root length and roots number under normal nitrogen (N^+^). The strongest phenotypic correlations were found between root dry weight and both root number and root length under N deficiency level, with the correlation coefficients of 0.647** and 0.616**, respectively. Simultaneously, there was a highly significant correlation coefficient between root length and root number with the value of 0.442** under N^−^ level. On the other hand, the significant correlation between root length and root dry weight was found with the value of 0.377* under N^+^ level.

### *qRDWN6*^*XB*^ mapping using a CSSL population

**Fig. 5:**
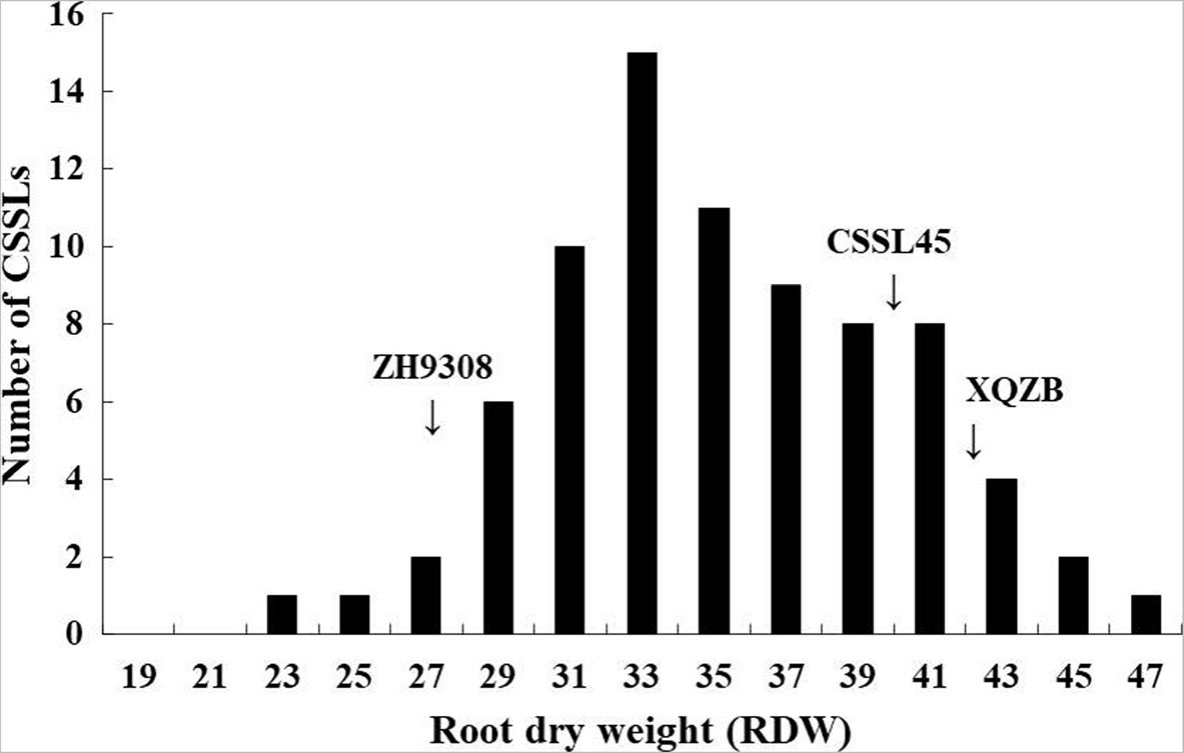
Frequency distribution of root dry weight (RDW) in CSSL population under nitrogen deficiency condition (N^−^). The arrows show average RDW of CSSL45, XQZB and ZH9308

**Fig. 6:**
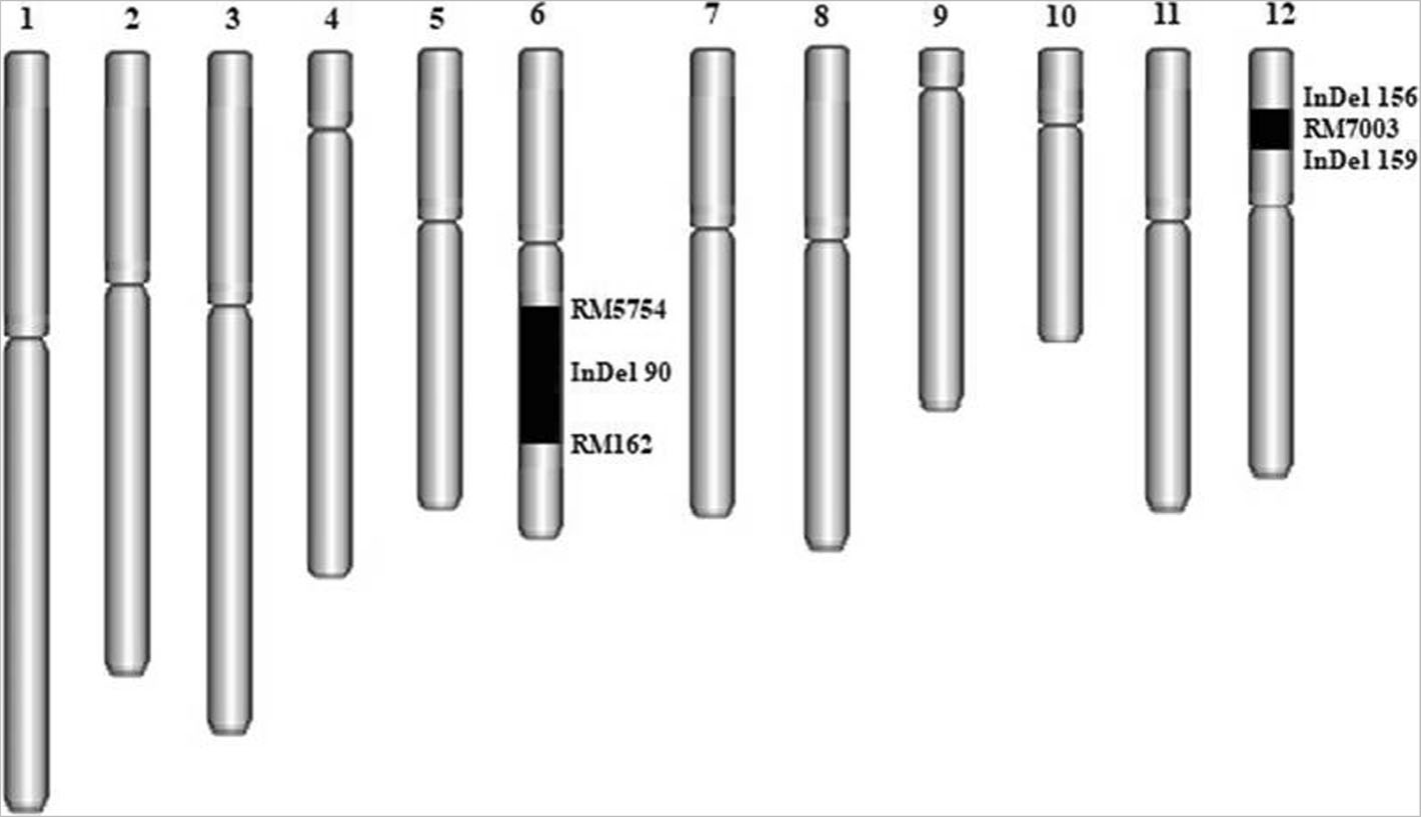
Graphical genotypes of CSSL45 derived from XQZB/ZH9308. *Black* bars denote the chromosome segments of parent XQZB. The marker name is shown on the right of chromosomes. Chromosomes numbers were showed above each chromosome

In our previous study and by using 75 CSSLs population derived from a cross between two parents (XieqingzaoB as a donor and Zhonghui9308 as a recipient) differ markedly for seedling root traits under N^−^ deficiency, *qRDWN6*^*XB*^ as a major QTL for root dry weight (RDW) against N-deficiency was detected. Using 120 simple sequence repeat (SSR) and insertion/deletion (InDel) markers, which were distributed along the whole rice genome according to previously reported linkage maps and known polymorphisms between the parents were used to identify the QTLs in CSSLs population based on a cut-off LOD value ⩾ 2.0. Analysis of variance based on genotype, treatment, and genotype x treatment interaction effects on root dry weight (RDW) revealed highly significant variance between the two N levels in the CSSL population (Table 2). RDW displayed a continuous variation and followed a normal frequency distribution under low N level (Fig. 5). This locus (named *qRDWN6*^*XB*^) detected in the region between RM5963 and RM20069 markers on the long arm of chromosome 6. It had the highest LOD score of 3.00, PVE% (phenotypic variance explained) of 16.84% and additive value of 6.14, which indicated that the *qRDWN6*^*XB*^ is likely the main effect QTL (Table 3). The positive allele from XieqingzaoB increased the root dry weight under N^−^ deficiency condition. One CSSL namely CSSL45, which had higher root dry weight values compared with Zhonghui9308 under N^−^ deficiency and harboring the *qRDWN6*^*XB*^, was selected for advanced investigation and their genotypes were shown in Fig. 6.

**Table 2:** Analysis of variance to determine the contribution to root dry weight (RDW) of genotype, treatment and their interaction in CSSLs population

Source of variance (S.O.V)	Degree of freedom (*df*)	Mean square (MS)	*F-*value (*F*)	*P-*value (*P*)
Genotype	75	43.795	362.30	**
Treatment ^a^	1	4291.216	86.55	**
Genotype x treatment	75	55.696	328.19	**
Residual	152	24.87		

^a^ Including two N levels (normal N^+^ and low N^−^)

**Significant at the 0.01 level

**Table 3:** Descriptive statistics for *qRDWN6*^*XB*^ mapped under low N in the CSSL population derived from XQZB and ZH9308

Trait	Nitrogen level	QTL	Chr.	Marker name	LOD score	PVE (%)	Add
RDW	N^−^	*qRDWN6* ^*XB*^	6	InD90	3.00	16.84	6.14

### Characterization of CSSL45 compared to ZH9308 under N^−^ deficiency

**Fig. 7:**
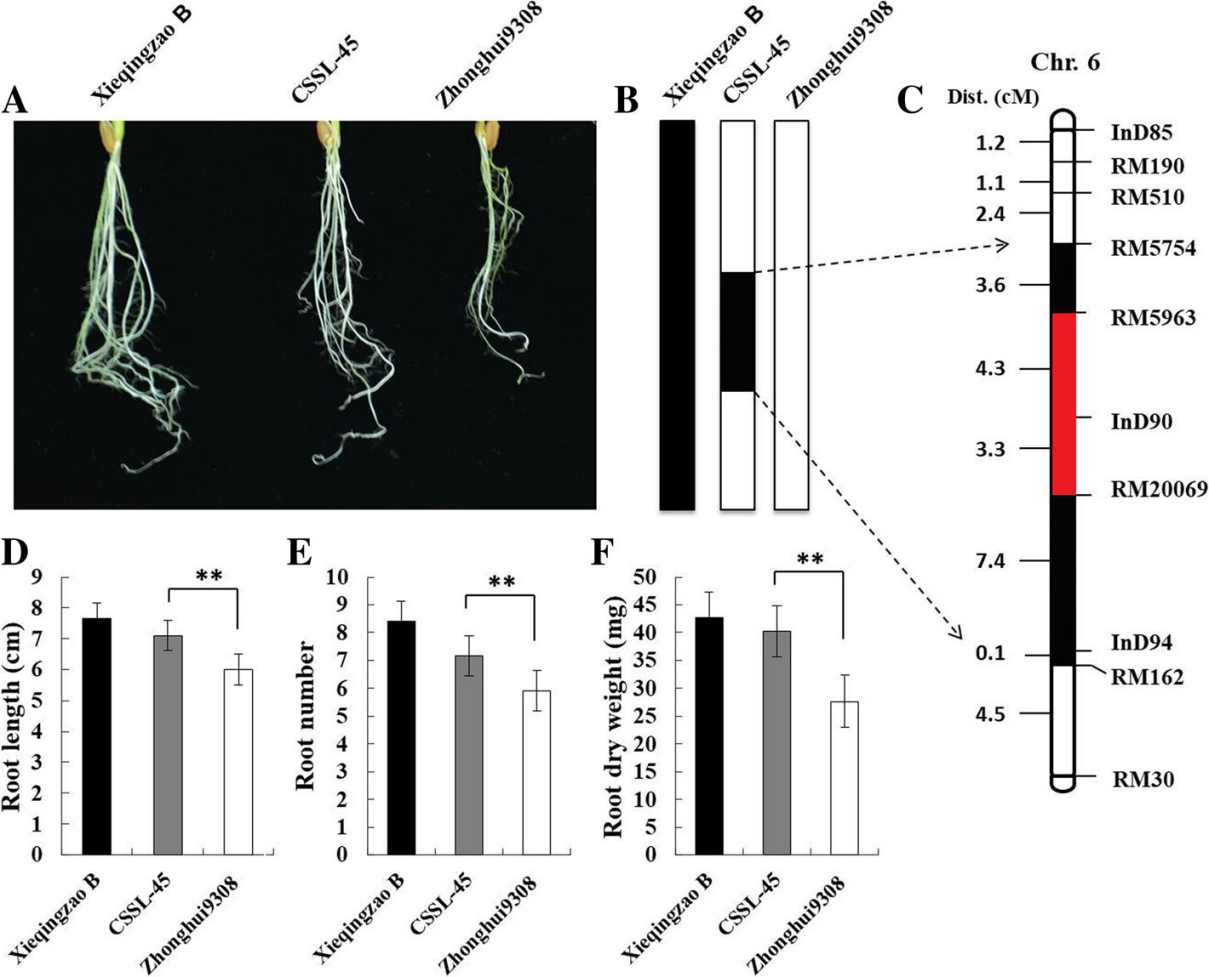
Phenotypic and genotypic performance of the parental lines. (**a**) Root morphology of CSSL45, XQZB and ZH9308 under N deficiency. (**b**) Graphical genotype of CSSL45 compared to the parental lines (chromosomal segments derived from XQZB in *black*). (**c**) Genetic location of *qRDWN6*^*XB*^ on chromosome 6 (maps to the interval in *Red*). (**d**, **e** and **f**) Root length, root number and root dry weight of CSSL45, XQZB and ZH9308 under N deficiency, respectively

**Fig. 8:**
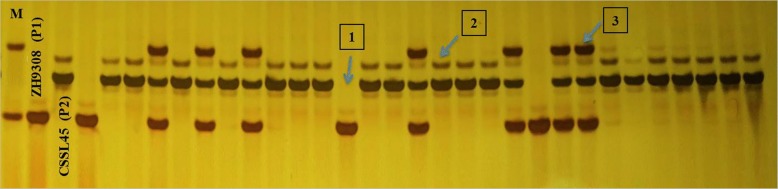
Genotypes of the parental lines ZH9308, CSSL45 and BC_5_F_2:3_ population using RM19807 marker. Number (1), indicate the genotypes of the homozygous ZH9308 allele, number (2) homozygous CSSL45 allele and number (3) heterozygous allele

To confirm the effect of *qRDWN6*^*XB*^ for root dry weight under N deficiency, one chromosome segment substitution line (CSSL) namely CSSL45, harboring this QTL and have the segment from XieqingzaoB between the markers RM5754 and RM162 (Fig. 7b, c), was crossed with the background parent Zhonghui9308. When Zhonghui9308 and CSSL45 in addition to XieqingzaoB were evaluated under N deficiency in hydroponic condition, the root dry weight in the CSSL45 was significantly higher than that of Zhonghui9308 (Fig. 7a, f). For other related root parameters and compared to Zhonghui9308, CSSL45 showed a longer root length and higher root number under low nitrogen (Fig. 7d, e). Therefore, the CSSL45 was backcrossed with the parent ‘Zhonghui9308’ and a total of 2200 F_2:3_ (BC_5_F_2:3_) seedlings obtained from the cross were screened for a fine mapping of *qRDWN6*^*XB*^ under N deficiency using the homozygous recombinant plants (Fig. 8).

### Fine mapping of the *qRDWN6*^*XB*^ to a 52.3-kb genomic region

**Fig. 9:**
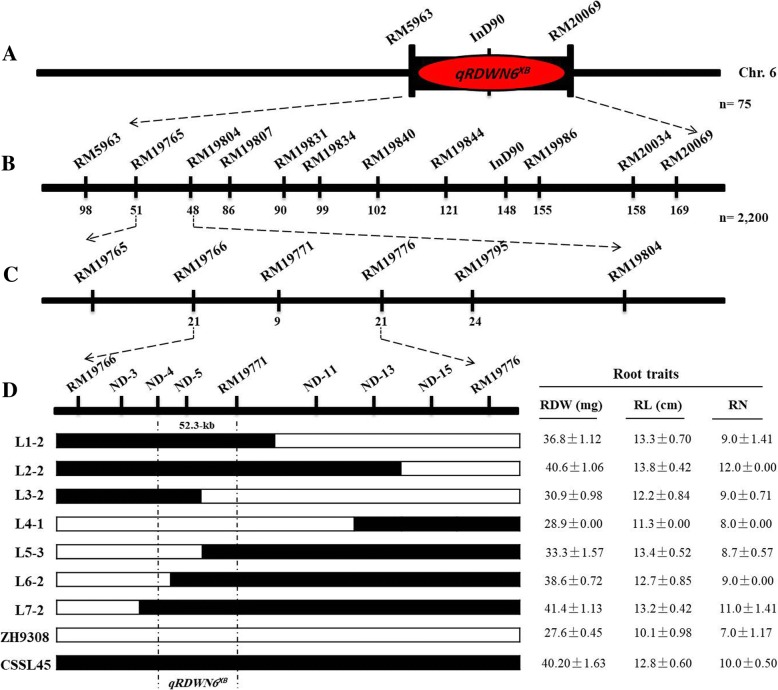
Mapping results of *qRDWN6*^*XB*^ on chromosome 6. (**a**) The genetic linkage map of the *qRDWN6*^*XB*^ based on 75 CSSLs. (**b**, **c**) the high resolution linkage map of the *qRDWN6*^XB^ region generated using BC_5_F_2:3_ population; the allele was mapped to the region between markers RM19766 and RM19776. (**d**) Genotypes and phenotypes of the parental lines (CSSL45 and ZH9308) and the homozygous recombinants plants for fine mapping. The *white* and *black* bars denote the marker genotype of ZH9308 and CSSL45, respectively. Root dry weight (RDW), root length (RL) and root number (RN) are presented as means ± SD

For genetic analysis and fine mapping of *qRDWN6*^*XB*^, a total of 2200 F_2:3_ individual plants derived from CSSL45/ZH9308 were screened and subjected to marker analysis by scanning RM5963 and RM20069 flanking *qRDWN6*^*XB*^ in the preliminary mapping (Fig. 9a). Out of 65 Simple Sequence Repeats (SSR) markers located in this genomic region (http:www.gramene.org/), nine markers were identified as polymorphic between the parents CSSL45 and ZH9308 (Fig. 9b). An analysis of RM5963 detected 98 recombination events between the marker and *qRDWN6*^*XB*^ on one side, while an analysis of RM20069 identified 169 recombination events between the marker and *qRDWN6*^*XB*^ on the other side. Simultaneously, the SSR markers RM20034, RM19986, InD90, RM19844, RM19840, RM19834, RM19831, RM19807 and RM19804 revealed 158, 155, 148, 121, 102, 99, 90, 86 and 48 recombinants, respectively, while RM19765 showed 51 recombinants on the other side (Fig. 9b). Therefore, the *qRDWN6*^*XB*^ was mapped to a 1003-kb genomic region between the interval RM19765 and RM19804. In this genomic region and out of 13 SSR markers, only four markers exhibited polymorphism between the two parents and used to narrow the region of *qRDWN6*^*XB*^ using the homozygous recombinant plants. The SSR markers RM19766, RM19771, and RM19776 were found to co-segregate with *qRDWN6*^*XB*^ and nine recombinant plants were revealed at RM19771, therefore *qRDWN6*^*XB*^ was mapped in the 247.3-kb interval of RM19766 to RM19776 (Fig. 9c). To narrow the targeted region of *qRDWN6*^*XB*^, fifteen new Insertion/Deletion (InDel) markers located at the substituted interval were designed and of which six markers exhibited polymorphism between the parents CSSL45 and ZH9308 (Table 1). Further recombinant screening with the newly designed Insertion/Deletion markers as well as RM19766, RM19771, and RM19776 showed that the gene was narrowed between ND-4 and RM19771 (Fig. 9d). With these newly designed markers and selected the homozygous recombinant plants, we evaluated the gene effect and then validated the phenotypic performance of root dry weight and two closely related traits, which varied from 28.9–41.4 mg for root dry weight, 11.3–13.8 cm for root length and 8–12 for root number. Finally and on the basis of the phenotypic and genetic analysis, the targeted region of *qRDWN6*^*XB*^ was localized to a 52.3-kb between the ND-4 and RM19771 markers (Fig. 9d).

### Candidate genes at the *RDWN6*^*XB*^ locus

Based on Rice Genome Annotation Project Database (http://rice.plantbiology.msu.edu/cgi-bin/gbrowse/rice/) and within the 8,981,538–9,033,871 bp in the target genomic region of *RDWN6*^*XB*^ on chromosome 6, there are nine predicted genes in this 52.3-kb region (Table 4). *LOC-Os06g15830* and *LOC_Os06g15890* expressed protein, *LOC_Os06g15840* and *LOC_Os06g15850* transposon protein, *LOC_Os06g15860, LOC_Os06g15870* and *LOC_Os06g15880* retrotransposon protein, *LOC_Os06g15900* conserved hypothetical protein and *LOC_Os06g15910* potassium transporter 24. *LOC_Os06g15910* has been reported previously as a major potassium transporter gene *OsHAK24*, so it was nominated and considered the most likely and strong candidate gene for the *RDWN6*^*XB*^ locus associated with nitrogen deficiency tolerance.

**Table 4:** Candidate genes located within 52.3-kb physical regions for *qRDWN6*^*XB*^ on chromosome 6

Gene ID	Gene start (bp)	Gene end (bp)	Putative function
*LOC-Os06g15830*	8,986,168	8,987,101	Expressed protein
*LOC_Os06g15840*	8,992,763	8,996,095	Transposon protein, putative, CACTA, En/Spm sub-class, expressed
*LOC_Os06g15850*	8,999,300	9,004,290	Transposon protein, putative, CACTA, En/Spm sub-class, expressed
*LOC_Os06g15860*	9,006,419	9,007,843	Retrotransposon protein, putative, unclassified, expressed
*LOC_Os06g15870*	9,009,481	9,014,615	Retrotransposon protein, putative, unclassified, expressed
*LOC_Os06g15880*	9,017,523	9,018,636	Retrotransposon protein, putative, unclassified
*LOC_Os06g15890*	9,019,348	9,019,788	Expressed protein
*LOC_Os06g15900*	9,021,414	9,022,376	Conserved hypothetical protein
*LOC_Os06g15910*	9,033,862	9,037,775	Potassium transporter 24, putative, expressed

## Discussion

Genetic populations played an important role in quantitative trait loci (QTLs) identifying and gene mapping. With different genetic segregating populations, such as F_2_ populations, chromosome segment substitution lines (CSSLs), double haploid lines (DH), near-isogenic lines (NILs) and recombinant inbred lines (RILs), hundreds genomic regions associated with several root-related traits under macro-nutrients deficiency have been identified in rice, but few have been fine mapped or cloned [38]. CSSLs have been a successful and effective tool for QTL mapping and positional cloning [39, 40]. Recently, there have been an increasing number of published studies on the genetic, molecular and and physiological regulation for root architecture as related to rice nutrient efficiency. A number of genes which are involved in changing the root development and root system architecture to facilitate enhanced nutrient acquisition have been identified in rice [33, 41, 42]. Two major QTLs, *TOND1*, confers tolerance to N deficiency tolerance in rice [33], *DEP1*, increases the tolerance to N deficiency [42], have been cloned. In our previous study, we analyzed the genomic regions in the XQZB/ZH9308 CSSLs population and found three putative QTLs under N deficiency during seedling stage using hydroponic culture. Among them, one strong QTL (*qRDWN6*^*XB*^) had the highest LOD value and that corresponds to the root dry weight was identified in the genomic region of RM5963-RM20069 on the long arm of chromosome 6 [24]. In the same chromosome, major QTLs for some root traits were also identified [19, 20, 26, 43] and affecting root system architecture, suggesting *qRDWN6*^*XB*^ might be a common genetic factor for root traits in various rice genotypes. Previous studies revealed that root dry weight (RDW) was significantly correlated with the other root traits under N deficiency, such as root number (RN), root length (RL), root thickness (RT) and root volume (RV), suggesting that these traits might be controlled and inherited by common genes [44, 45]. Root dry weight has been considered the most important component associated with crop yields than the other root traits. Therefore, significantly correlations analysis between root dry weight (RDW) and other related root traits like root number (RN) and root length (RL) under N deficiency were observed (Fig. 4). In this study, we delimited the chromosome segment containing *qRDWN6*^*XB*^ and fine mapped to a 52.3-kb for the first time. One CSSL, CSSL45-*qRDWN6*^*XB*^, which consistently produced higher root dry weight was selected for advanced study and crossed with the genetic background parent Zhonghui9308 and a total of 2200 F_2:3_ (BC_5_F_2:3_) seedlings obtained from the cross were screened for a fine mapping of *qRDWN6*^*XB*^ under N^−^ deficiency condition. In addition, CSSL45 as a line harboring this locus was significantly higher than Zhonghui9308 in root dry weight (RDW) under two nitrogen levels (N^+^ and N^−^), indicating *qRDWN6*^*XB*^ could stably express in different conditions and there were more CSSLs with high RDW under N^−^ deficiency than that of the two parents (Fig. 5). This transgressive segregation of root traits including RDW under different N treatments was also observed in introgression lines [26] and recombinant inbred line (RIL) population [28].

In rice crop, the important functions of the root system are the responses to low N availability and mainly through enhanced root traits like root number, root length, root density and root thickness to absorb water and primarily nutrients [23, 46–48]. Root dry weight is better related to crop yields and important measurement because it is a summary of all root traits. In this investigation, we identified *qRDWN6*^*XB*^*,* a major QTL enhancing tolerance of N deficiency in rice. This QTL is the first locus fine mapped for root dry weight under N deficiency in the target region 8,981,538 - 9,033,871 bp on chromosome 6. Numerous studies have identified several QTLs/genes for nitrogen use efficiency on the same chromosome. For example, Song et al. [49] identified *AspAT3* gene within the 23,738,029 - 23,738,154 bp region, which is contributed to nitrogen and carbon metabolism in rice. Around the waxy gene on chromosome 6, Shan et al. [50] mapped *qNUEp-6* as a major QTL for nitrogen utilization in the 1.76–2.09 Mb regions. Under normal and low nitrogen conditions and within the 28.13–29.63 Mb regions, Tong et al. [22] identified QTLs associated with dry weight and grain yield on the chromosome 6. Using rice chromosome segment substitution lines (CSSLs) a novel QTL of effective panicle and yield in the range of 2.29–2.83 Mb was detected by Wang et al. [51]. Also, a novel locus related to NUE was identified using a genome-wide association analysis of 184 rice genotypes at 4,845,258 - 4,845,375 bp and near the SSR marker RM314 [52]. Yang et al. [53] delimited the *qNUE6* locus in 8,647,275 - 8,913,783 bp region. However, these QTLs/genes are not the same loci as *qRDWN6*^*XB*^; therefore, this QTL is a newly discovered locus controlling nitrogen deficiency tolerance in the target region. Among the nine predicted genes in the fine-mapped region 52.3-kb, only one gene *LOC_Os06g15910* has been reported previously as a major potassium transporter gene *OsHAK24* [54], it was considered the most likely and strong gene for the *RDWN6*^*XB*^ locus and might be the candidate gene responsible for nitrogen deficiency tolerance. Previous studies have demonstrated different genes controlling nitrogen use efficiency and nitrogen deficiency tolerance in rice. In the concentrations of low and high nitrate ions, two genes *NRT1.1B* had a nitrate-transporting activity and *NRT2* a high-affinity transporter were found by Hu et al. [55] but *NRT2* can’t transfer NO^− 3^ independently. *OsNRT2.3b* able to increase the uptake of nitrogen and phosphorus, nitrogen use efficiency and improving the grain yield [56]. At different levels of nitrate, Yan et al. [57] showed that *OsNAR2.1* is able to interact with *OsNRT2–1, OsNRT2–2*, and *OsNRT2–3* genes, and can enhance nitrate uptake by rice roots at different levels of nitrate. *TOND1* as a major QTL was detected on chromosome 12 and its over-expression enhanced the nitrogen deficiency tolerance in rice [33]. *OsAMT1–1, OsAMT1–2,* and *OsAMT1–3* are the major three ammonium transporter genes were identified in rice and played a pivotal role in absorbing NH^+ 4^ [58–60]. These results have greatly enhanced our understanding of the regulation of nitrogen use efficiency and nitrogen deficiency tolerance in rice. Here, we report the characterization and identification of a novel QTL, *qRDWN6*^*XB*^, that regulates root dry weight in rice to a region of 52.3-kb (Fig. 9d), and identified nine predicted genes as viable candidates for it. One of them, *LOC_Os06g15910* previously reported as a major potassium transporter gene *OsHAK24* [54] might play an important role in nitrogen deficiency tolerance in rice. The annual consumption of N fertilizers increases year after year, causing severe pollution to agricultural and ecological environments. Only 30–45% of N fertilizer is efficiently used by rice plants [61]. Therefore, the rice cultivars harbor the *qRDWN6*^*XB*^ may significantly decrease the use of N fertilizers, reduce the cost of rice production, alleviate pollution and protect the environment. *qRDWN6*^*XB*^ is expected to have wide use potential in rice breeding programs and will play an important role in the ‘Second Green Revolution’.

## Conclusions

In summary, a major QTL, *qRDWN6*^*XB*^ was identified in the rice response to N deficiency and found that it enhances a positive regulator of root system architecture. Based on the target CSSL and homozygous recombinant population, *qRDWN6*^*XB*^ was delimited to a 52.3-kb region on chromosome 6. Nine candidate genes were identified in the target region using gene annotation information. *LOC_Os06g15910* previously reported as a potassium transporter gene in this region and might play an important role in nitrogen deficiency tolerance of rice. The identification of *qRDWN6*^*XB*^ provides a new genetic resource for breeding rice varieties and a starting point to improve grain yield despite the decreased input of N fertilizers. The newly developed and tightly linked InDel marker ND-4 will be useful to improve the root system architecture by marker-assisted selection (MAS) in rice breeding programs. But to understand how *RDWN6*^*XB*^ affects the agronomic characteristics of rice, further study is needed to clarify their molecular and biological functions through cloning and transgenic approaches.

## Abbreviations

CSSLs: Chromosome Segment Substitution Lines
DH: Double haploid lines
InDel: Insertion/deletion
LOD: Logarithm of the odds
MAS: Marker assisted selection
N^−^: Low nitrogen
N^+^: Normal nitrogen
NILs: Near-isogenic lines
PVE: Phenotypic variance explained
QTL: Quantitative trait loci
RDW: Root dry weight
RDWN: Root dry weight under N deficiency
RILs: Recombinant inbred lines
RL: Root length
RN: Root Number
SSR: Simple Sequence Repeat
XQZB: XieqingzaoB
ZH9308: Zhonghui9308
